# Expression of the Stem Cell Factor Nestin in Malignant Pleural Mesothelioma Is Associated with Poor Prognosis

**DOI:** 10.1371/journal.pone.0139312

**Published:** 2015-09-30

**Authors:** Svenja Thies, Martina Friess, Lukas Frischknecht, Dimitri Korol, Emanuela Felley-Bosco, Rolf Stahel, Bart Vrugt, Walter Weder, Isabelle Opitz, Alex Soltermann

**Affiliations:** 1 Institute of Surgical Pathology, University Hospital Zurich, Zurich, Switzerland; 2 Division of Thoracic Surgery, University Hospital Zurich, Zurich, Switzerland; 3 Cancer Registry, University Hospital Zurich, Zurich, Switzerland; 4 Clinic of Oncology, University Hospital Zurich, Zurich, Switzerland; Virginia Commonwealth University, UNITED STATES

## Abstract

**Background:**

The epithelioid and sarcomatoid histologic variants of malignant pleural mesothelioma (MPM) can be considered as E- and M-parts of the epithelial-mesenchymal transition (EMT) axis; the biphasic being an intermediate. EMT is associated with an increase of stem cell (SC) traits. We correlated the neural crest SC marker nestin and the EMT marker periostin with histology, type of neo-adjuvant chemotherapy (CT) and overall survival (OS) of MPM patients.

**Patients and Methods:**

Tumor tissues of a historic cohort 1 (320 patients) and an intended induction chemotherapy followed by extrapleural pneumonectomy (EPP) cohort 2 (145 patients) were immunohistochemically H-scored (intensity of immunoreactivity multiplied by frequency of stained cells). Paired chemo-naïve biopsies and -treated surgical specimens were available for 105/145 patients. CT included platinum/gemcitabine (Pla/Gem) or platinum/pemetrexed (Pla/Pem).

**Results:**

Expression of any cytosolic nestin progressively increased from epithelioid to biphasic to sarcomatoid MPM in cohort 1, whereas the diagnostic markers calretinin and podoplanin decreased. In cohort 2, Pla/Pem CT increased the expression level of nestin in comparison to Pla/Gem, whereas the opposite was found for periostin. In Pla/Pem treated patients, nestin was higher in biphasic MPM compared to epithelioid. In addition to non-epithelioid histology, any expression of nestin in chemo-naïve biopsies (median overall survival: 22 vs. 17 months) and chemo-treated surgical specimens (18 vs. 12 months) as well as high periostin in biopsies (23 vs. 15 months) were associated with poor prognosis. In the multivariate survival analysis, any nestin expression in chemo-naïve biopsies proved to be an independent prognosticator against histology. In both pre- and post-CT situations, the combination of nestin or periostin expression with non-epithelioid histology was particularly/ dismal (all p-values <0.05).

**Conclusions:**

The SC marker nestin and the EMT marker periostin allow for further prognostic stratification among histologic variants of MPM. Their expression level is influenced by neo-adjuvant chemotherapy.

## Introduction

Malignant pleural mesothelioma (MPM) is a highly aggressive, asbestos-related neoplasm characterized by rapid and diffuse local growth, late metastases, and early death. MPM elaborates the two distinct histologic variants epithelioid and sarcomatoid. The mixture of both is called biphasic.

Mesothelial cells are derived from the coelomic cavity lining cells which originate from mesodermal mesenchyme via mesenchymal-epithelial transition (MET), leading to co-expression of the intermediate filaments cytokeratin and vimentin. The opposite epithelial-mesenchymal transition (EMT) from epithelioid to sarcomatoid mesothelioma in turn reflects a reversion of embryological development and is associated with increased tumor aggressiveness and poor survival, respectively. During this transdifferentiation, all relevant diagnostic mesothelioma markers such as calretinin, podoplanin detected by the D2-40 antibody, WT1 and CK5/6 decrease. Previously, we found a calretinin expression in 91% of epithelioid and 57% of sarcomatoid tumors, respectively. D2-40 immunoreactivity was present in 66% of epithelioid and 30% of sarcomatoid [1]. Few data are available on markers that increase towards sarcomatoid differentiation or allow for further stratification of prognosis among histologic variants.

EMT has been shown to confer stem cell (SC) traits to tumor cells [2] and one therefore expects a higher expression of EMT/SC markers in sarcomatoid tumors. We investigated the well-recognized EMT marker N-glycoprotein periostin, both tumor-cell associated and stromal [3–4] and found it to be associated with sarcomatoid MPM [5]. Periostin, tenascin and osteopontin are the main non-structural secreted matricellular proteins which form a key component of both desmoplastic tumor stroma and granulation tissue [6]. In asbestos-exposed persons, serum osteopontin levels were shown to be different in cancer-free versus MPM patients [7].

The intermediate filament protein nestin is a neuroectodermal stem/progenitor cell marker [8–9]. Self-renewing melanoma progenitor cells expressed nestin [10] and both intermediate filament proteins nestin and vimentin were found to colocalize in melanoma [11]. Next to neural cells and melanocytes, the neural crest also generates mesenchymal cell types and nestin was detected in mesodermal cells like fibroblasts or hepatic stellate cells [12–13]. In the kidney, nestin-positive tubular cells co-expressed vimentin, suggesting these cells reverted to a mesenchymal phenotype [14] and Nes-Cre1-mediated recombination occurred in progenitor cell types of the developing mesonephros [8]. Finally, nestin positive mesenchymal stem cells (MSCs) were found to constitute an essential hematopoietic stem cell (HSC) niche component in the bone marrow [15].

Recent work on nestin, mesothelin and epithelial membrane antigen (EMA) expression in developing and adult serous membranes indicated that superficial pleural mesothelial cells are always nestin negative, whereas in the submesothelial layer cells nestin expression decreases during development. Nestin protein expression was immunohistochemically detected in 13% of the tumor cells of 6 epithelioid mesotheliomas [16].

Herein, we addressed the hypothesis that expression of the SC marker nestin or the EMT marker periostin may stratify with the histologic variant and the overall survival (OS) of MPM patients. Further, we investigated the effect of neo-adjuvant chemotherapy (CT) using platinum/gemcitabine (Pla/Gem) or platinum/pemetrexed (Pla/Pem) on the expression of these markers. A historic test cohort 1 of 320 patients and a cohort 2 consisting of 145 patients with intention-to-treat (ITT) by induction chemotherapy followed by extrapleural pneumonectomy were investigated by IHC on tissue microarrays. The ITT patient cohort 2 included paired chemo-naïve diagnostic biopsies and chemo-treated surgical specimens.

## Materials and Methods

### Patient cohorts and MPM histology

Two MPM patient cohorts were set up. Cohort 1 ranged from 1975 to 2004 and comprised 320 patients from whom mesothelioma and lung tissue was sent to the Zurich Pneumoconiosis Research Group for mineralogical assessment of dust exposure by TEM-EDX (transmission electron microscopy, energy dispersive X-ray spectroscopy). This cohort consisted mostly of autopsy cases with only limited clinical data. Cohort 2 spans the decade of 1999 to 2009 and includes 145 non-redundant MPM patients referred to the University Hospital Zurich for intended multimodality treatment with induction chemotherapy followed by extrapleural pneumonectomy (EPP). Paraffin blocks of pre-chemotherapy (CT) diagnostic pleural biopsies were gathered from all Institutes of Pathology of referral hospitals. All cases were entirely reviewed for histologic classification on both whole sections used for the sign-out and TMA cores. MPM was considered biphasic if the minor moiety exceeded 10% according to the WHO guidelines. Written informed consent was given by the patients and documented on the clinical information system at the time of surgery. The study was approved by the Ethical Commission of the Canton of Zurich under reference number KEK ZH-Nr. 29-2009/14.

### Tissue microarray construction and immunohistochemistry

MPM TMAs were constructed as previously described (1). Four (surgical specimens) or two (diagnostic biopsies) tissue cores of 0.6 mm diameter were taken from 1 or 2 most representative tumor blocks and transferred into the recipient paraffin block. Concerning the biphasic variant, cores were punched out of an area considered to be biphasic on the corresponding HE (hematoxylin-eosin) whole section. In total, 7 TMAs were manufactured. TMA sections were stained on an automated IHC platform (Ventana Medical System, Tucson, AZ, USA) with the Mab anti-nestin (clone 10C2, 1:100 dilution, Chemicon International Inc., Temecula, CA, USA). Detection was performed with respective secondary antibody and Ultraview Amp kit (Ventana). Staining of periostin, calretinin and podoplanin (D2-40 antibody) was performed as previously described [1,5].

### Cell blocks

The biphasic MPM cell line SPC212 and the immortalized mesothelial cell line MET5A were grown in RPMI 1640, supplemented with 10% fetal bovine serum. Cells were centrifuged at 2000 x g for 10 min at room temperature, clotted by addition of plasma (4 droplets) and thrombin (1 droplet) and processed as formalin-fixed paraffin-embedded (FFPE) cell blocks as described [17].

### Data interpretation and statistical analysis

The intensity of tumor-associated cytosolic immunoreactivity was scored semi-quantitatively 0 (negative), 1 (weak), 2 (moderate) or 3 (strong). The frequency of stained tumor cells was scored 0, 10 (1–10%), 50 (11–50%) or 100 (51–100%). The product of intensity of immunoreactivity and frequency of stained cells was called H-score (I x F, range 0–300). In order to get an H-score per patient the 4 cores or the 2 cores were summed up and divided by 4 or 2, respectively. Non-parametric tests were used to evaluate the association of H-scores and histology (Mann-Whitney U test). To compare the change of expression in paired chemo-naïve and -treated samples the Wilcoxon signed rank test was used. Tumor response to chemotherapy was evaluated based on modified Response Evaluation Criteria in Solid Tumors (RECIST) [18]. The association of modified RECIST criteria and H-scores was tested with the Kruskal-Wallis test for independent samples. For survival analyses the H-score was dichotomized closest to the median. To analyse the effect of marker expression and histology on survival, 4 groups were made with low/high marker expression and epithelioid/non-epithelioid histology. The Kaplan-Meier method was applied using log rank tests. Patients having a survival ≤ 1 month post-surgery were excluded. Overall survival (OS) represents survival from the date of diagnosis to the time-point of death for diagnostic biopsies and from the date of surgery to the time point of death for surgical specimens. If no event occurred the OS was calculated until last follow-up and the cases were censored in the analysis. For multivariate analysis significant parameters were introduced into a Cox regression model, using histology as categorical variable. P-values <0.05 were considered significant. All statistical analyses were performed on SPSS version 22 software (SPSS Inc., Chicago, USA).

## Results

### Cohort description

Table 1 summarizes the clinico-pathologic data of the 2 cohorts: In total, tumor tissue of 465 MPM patients was analysed. The median overall survival (OS) of cohort 2 was 18 months (95% confidence interval (CI): 13–23 months) and 98% of the patients deceased during the observation time. All cohort 2 patients were intended to be treated with induction chemotherapy followed by EPP and therefore received 3 cycles of Pla/Gem (n = 59) or Pla/Pem (n = 85). One patient received another regimen. EPP was thereafter performed in 107 (74%) of patients, the others underwent pleurectomy or thoracotomy or had no surgery.

**Table 1:** Summary of clinico-pathological and immunohistochemical data.

		Cohort 1	Cohort 2	
**N patients**		320	145	
**Time period**		1975–2004	1999–2009	
**Intervention**	EPP		107 (74%)	
	Pleurectomy		8 (5%)	
	Thoracotomy		16 (11%)	
	No surgery		14 (10%)	
	Any Surgery	57 (18%)		
	Autopsy	263 (82%)		
RECIST*	Partial response (PR)		29 (30%)	
	Stable disease (SD)		42 (43%)	
	Progressive disease (PD)		26 (27%)	
**Median age (range)**		64 (32–95)	61 (36–72)	
**Sex**	male	303 (95%)	133 (92%)	
	female	17 (5%)	12 (8%)	
			**pre CT**	**post CT**
			118 patients	133 patients
**Histology**	epithelioid	129 (40%)	75 (64%)	73 (55%)
	biphasic	144 (45%)	38 (32%)	52 (39%)
	sarcomatoid	47 (15%)	5 (4%)	8 (6%)
**Nestin H-score**	0	197 (62%)	66 (65%)	72 (59%)
	1–100	82 (26%)	27 (26%)	42 (34%)
	101–200	24 (7%)	6 (6%)	5 (4%)
	201–300	17 (5%)	3 (3%)	3 (3%)
**Periostin H-score**	0		9 (8%)	
	1–100		84 (71%)	82 (62%)
	101–200		19 (16%)	31 (24%)
	201–300		6 (5%)	19 (14%)

In cohort 2, the distribution of immunohistochemical H-scores (intensity of immunoreactivity multiplied by frequency of stained cells) is indicated for both pre-and post-chemotherapy (pre-CT/post-CT) biopsies and surgical specimens, respectively. ^a^modified RECIST criteria were only available for 97 patients.

### Nestin and periostin expression in MPM cohort 1 and 2

**Fig 1:**
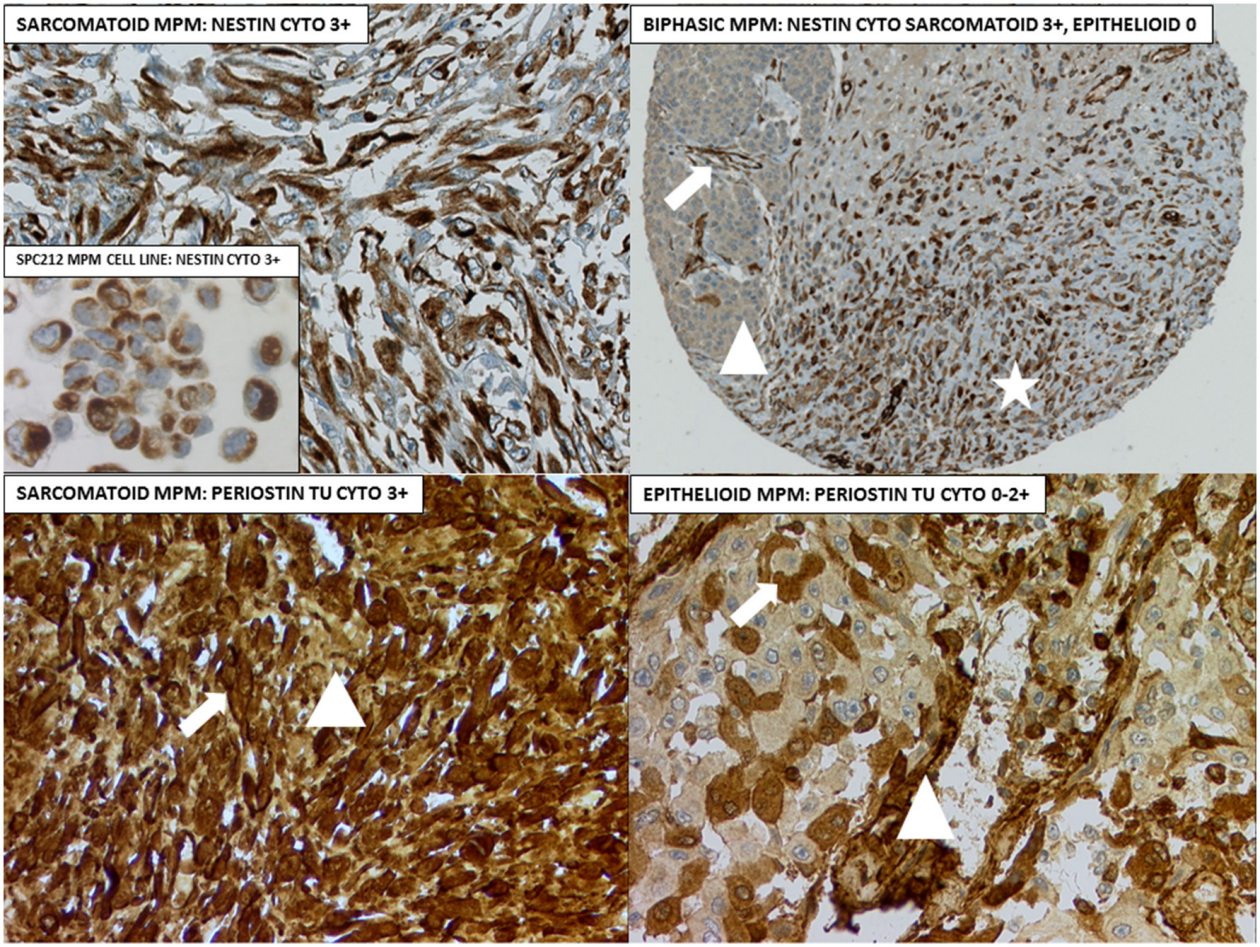
IHC examples for nestin and periostin. Upper left: Sarcomatoid MPM and the biphasic mesothelioma cell line SPC212 (insert). Upper right: Biphasic MPM tissue core with >10% epithelioid moiety (triangle) and predominant sarcomatoid differentiation (asterisk). Arrow = nestin-positive vessel endothelia. Lower left: Arrow = tumor cell-associated cytosolic periostin, triangle = stromal periostin. Lower right: Arrow = tumor cell-associated periostin, triangle = stromal periostin along vessel structures. Original magnifications 100x (upper right), 200x (other 3 panels) and400x (insert).

Robust nestin immunoreactivity was observed in the biphasic mesothelioma cell line SPC212 and the MPM tumor tissues (Fig 1) as well as in the immortalized mesothelial cell line MET5A (data not shown). Both the intermediate filament protein nestin and the tumor cell-associated N-glycoprotein periostin were expressed in the cytosol. The nestin and periostin H-score ranges are shown in Table 1. Thus, any nestin expression was found in 38 to 41% of MPM in both cohorts. In the pre-chemotherapy biopsies of cohort 2, 8% of tumors were completely negative for periostin. The majority displayed a weak cytosolic expression with H-score up to 100.

### Correlation of nestin and diagnostic epithelioid markers with histology in cohort 1

**Fig 2:**
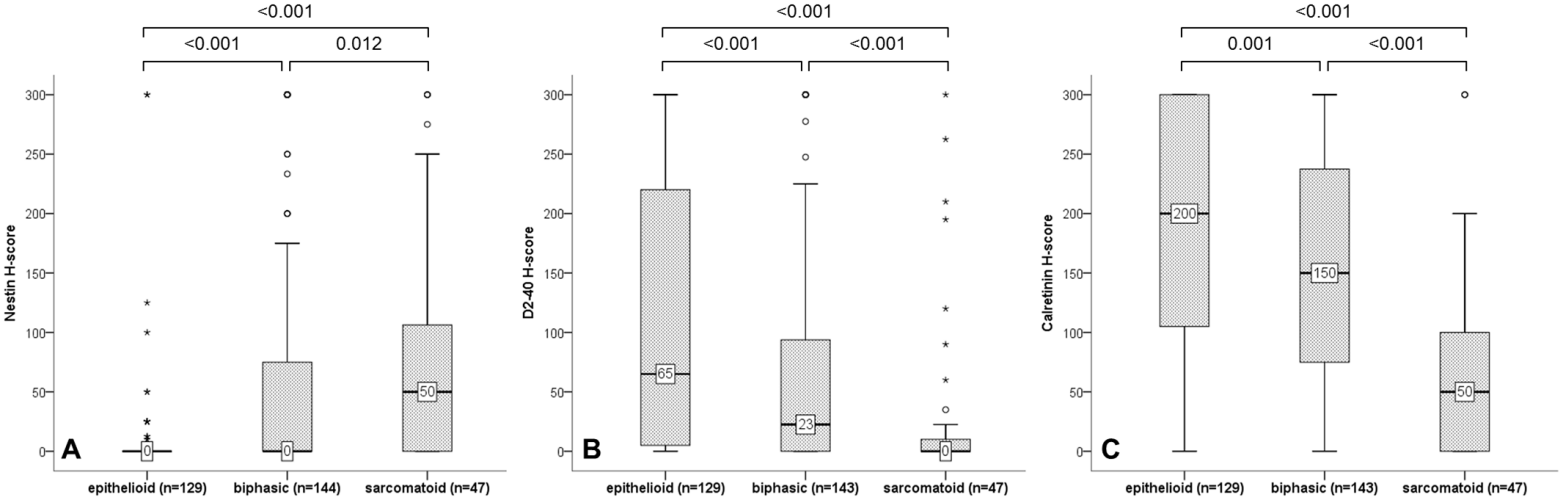
Association of markers with histology in cohort 1. H-scores of nestin (A) were significantly higher in sarcomatoid MPM whereas the diagnostic epithelioid markers podoplanin/D2-40 (B) and calretinin (C) were significantly decreased in sarcomatoid MPM; Mann-Whitney U tests.

In cohort 1, nestin immunoreactivity increased progressively from epithelioid (median: 0 interquartile range (IQR): 0–0) to biphasic (median: 0, IQR: 0–100) to sarcomatoid MPM (median 50, IQR: 0–200), whereby all transitions including epithelioid-sarcomatoid, epithelioid-biphasic and biphasic-sarcomatoid were significant. The opposite was observed for the diagnostic epithelioid markers calretinin and podoplanin, recognized by the D2-40 antibody (Fig 2).

### Correlation of nestin and periostin with neo-adjuvant chemotherapy and histology in cohort 2

In cohort 2, the protein expression levels of periostin but not nestin significantly increased after neo-adjuvant chemotherapy (Table 2). When comparing the two neo-adjuvant regimens, we observed that the expression of periostin was significantly higher after Pla/Gem compared to Pla/Pem. The opposite was found for nestin: Its immunoreactivity was significantly lower after Pla/Gem compared to Pla/Pem.

**Table 2:** Change of nestin and periostin expression after neo-adjuvant chemotherapy.

	Nestin H-score			Periostin H-score		
	median (IQR)		p	median (IQR)		p
Neo-adj. CT	Chemo -	Chemo +		Chemo -	Chemo +	
	0 (0–10)	0 (0–10)	n.s.	20 (10–100)	75 (15–150)	*0*.*002*
CT regimen	Pla/Gem	Pla/Pem		Pla/Gem	Pla/Pem	
	0 (0–0)	3 (0–10)	*0*.*024*	110 (20–188)	33 (13–150)	*0*.*027*

Neo-adj. CT: Comparison of nestin and periostin H-scores in chemo-naïve biopsies (Chemo-) with surgical specimens after neo-adjuvant chemotherapy (Chemo +) (paired samples Wilcoxon signed rank test). CT regimen: Comparison of marker expression after application of two different chemotherapy regimens: Pla/Gem, platinum + gemcitabine versus Pla/Pem, platinum + pemetrexed (Mann-Whitney U test for independent samples). IQR: interquartile range; CT: chemotherapy.

**Fig 3:**
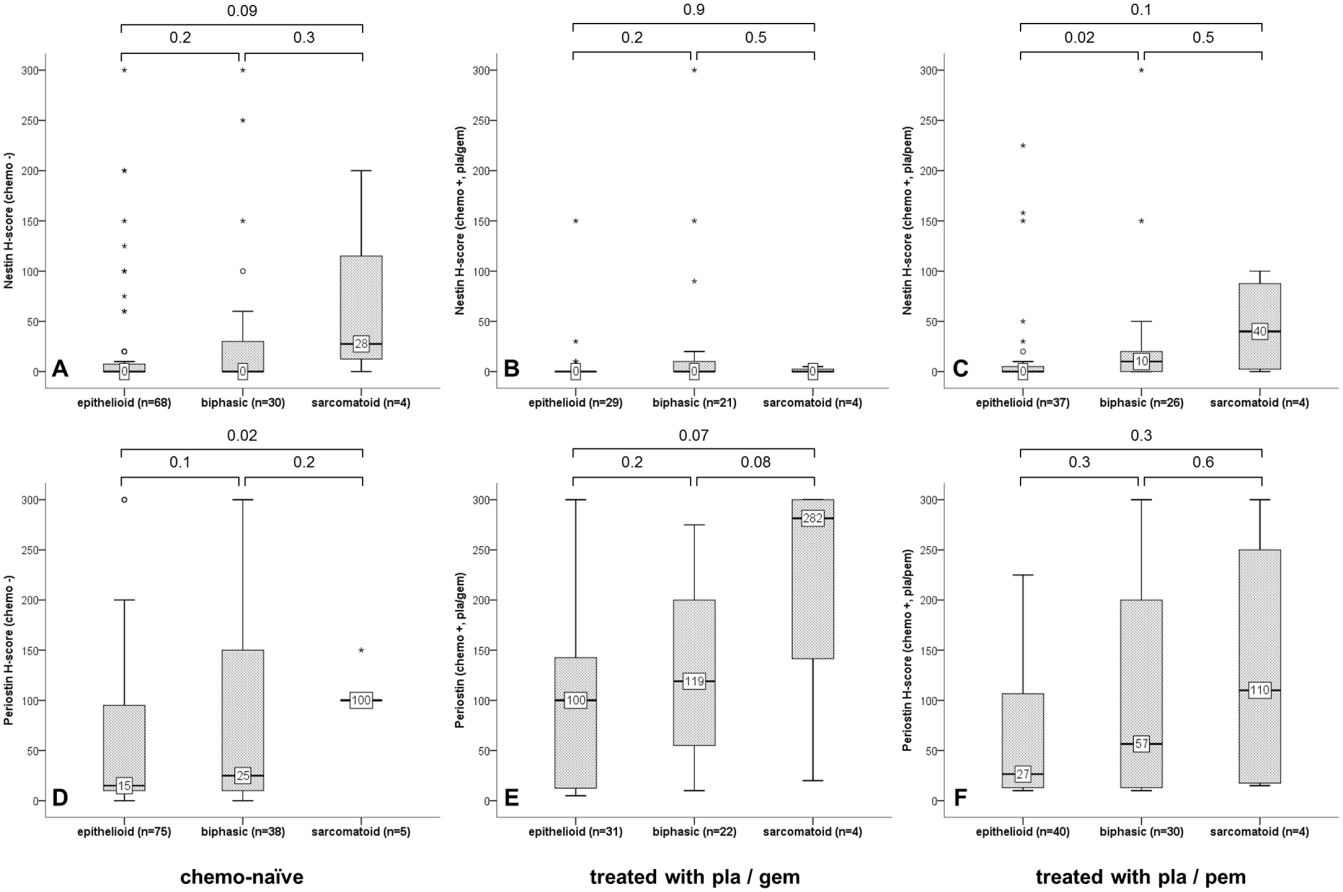
Associations of markers with histology according to neo-adjuvant CT. Box plots of nestin (A-C) and periostin (D-F) expression correlated with histologic variant in cohort 2 in chemo-naïve biopsies (A/D) and surgical specimens treated with with Pla/Gem (B/E) or Pla/Pem (C/F); Mann-Whitney U tests.

Itemization of neo-adjuvant chemotherapy according to histology (Fig 3) yielded following results: A significant increased nestin protein expression was observed in Pla/Pem treated biphasic MPM in comparison to epithelioid (Fig 3C). Periostin protein expression was higher in chemo-naïve sarcomatoid MPM compared to epithelioid (Fig 3D). No significant different expression among histologic variants was observed after Pla/Gem or Pla/Pem chemotherapy.

No significant association between responses to induction chemotherapy (partial response vs. stable disease vs. progressive disease) and the nestin as well as periostin H-scores before and after chemotherapy could be detected. There was also no significant association between response and H-score when the Pla/Gem and Pla/Pem treated patients were analysed separately.

### Association with overall survival

As shown in Table 3, epithelioid histology was associated with longer OS compared to non-epithelioid in chemo-naïve and chemo-treated samples. High expression of periostin in chemo-naïve samples was associated with shorter OS. Expression of nestin in chemo-naïve as well as chemo-treated samples was also associated with decreased OS. Combination of histology with marker expression gave following results: Patients with an epithelioid MPM that showed no expression of nestin in the untreated sampled showed the longest median OS (24 months) whereas patients with non-epithelioid MPM and expression of nestin had the shortest OS (10 months) of the 4 groups. Patients with a non-epithelioid MPM and high periostin levels in chemo-naïve samples had a significantly worse median OS (11 months) than the other 3 groups (median OS 22–25 months). In chemo-treated surgical specimens, patients with non-epithelioid MPM and expression of nestin or high expression of periostin had a shortened median OS.

**Table 3:** Univariate analysis of overall survival.

		n	Median OS	95% CI	p
**Chemo-naïve samples**					
Histology	epithelioid	74	23	20–26	*0*.*016*
	non-epithelioid	39	14	10–18	
Nestin	no expression	64	22	17–27	*0*.*017*
	expression	33	17	9–24	
Periostin	low H-score (≤ 20)	62	23	20–26	*0*.*043*
	high H-score (> 20)	51	15	9–21	
Histology + nestin	no nestin expression + epithelioid	47	24	17–31	*0*.*002*
	no nestin expression + non-epithelioid	17	15	11–19	
	nestin expression + epithelioid	20	19	11–28	
	nestin expression + non-epithelioid	13	10	7–13	
Histology + periostin	low periostin H-score + epithelioid	46	22	19–25	*0*.*001*
	low periostin H-score + non-epithelioid	16	25	22–28	
	high periostin H-score + epithelioid	28	23	18–29	
	high periostin H-score + non-epithelioid	23	11	6–16	
**Chemo-treated samples**					
Histology	epithelioid	70	19	15–24	*0*.*003*
	non-epithelioid	55	11	9–14	
Nestin	no expression	67	18	14–22	*0*.*038*
	expression	47	12	2–21	
Periostin	low H-score (≤ 75)	63	19	17–21	n.s.
	high H-score (> 75)	61	16	10–22	
Histology + nestin	no nestin expression + epithelioid	43	22	13–30	*0*.*008*
	no nestin expression + non-epithelioid	24	11	8–15	
	nestin expression + epithelioid	21	18	13–23	
	nestin expression + non-epithelioid	26	7	3–12	
Histology + periostin	low periostin H-score + epithelioid	38	19	14–24	*0*.*005*
	low periostin H-score + non-epithelioid	25	14	5–23	
	high periostin H-score + epithelioid	31	18	12–25	
	high periostin H-score + non-epithelioid	30	10	6–14	

Association of histologic variant as well as nestin and periostin H-scores (alone or in combination) in chemo-naïve biopsies and chemo-treated surgical specimens with patient’s overall survival (OS) indicated. Median OS was calculated in months from the date of diagnosis for chemo-naïve and from the date of surgery for chemo-treated samples. P-value was determined by log rank test. CI: confidence interval.

**Fig 4:**
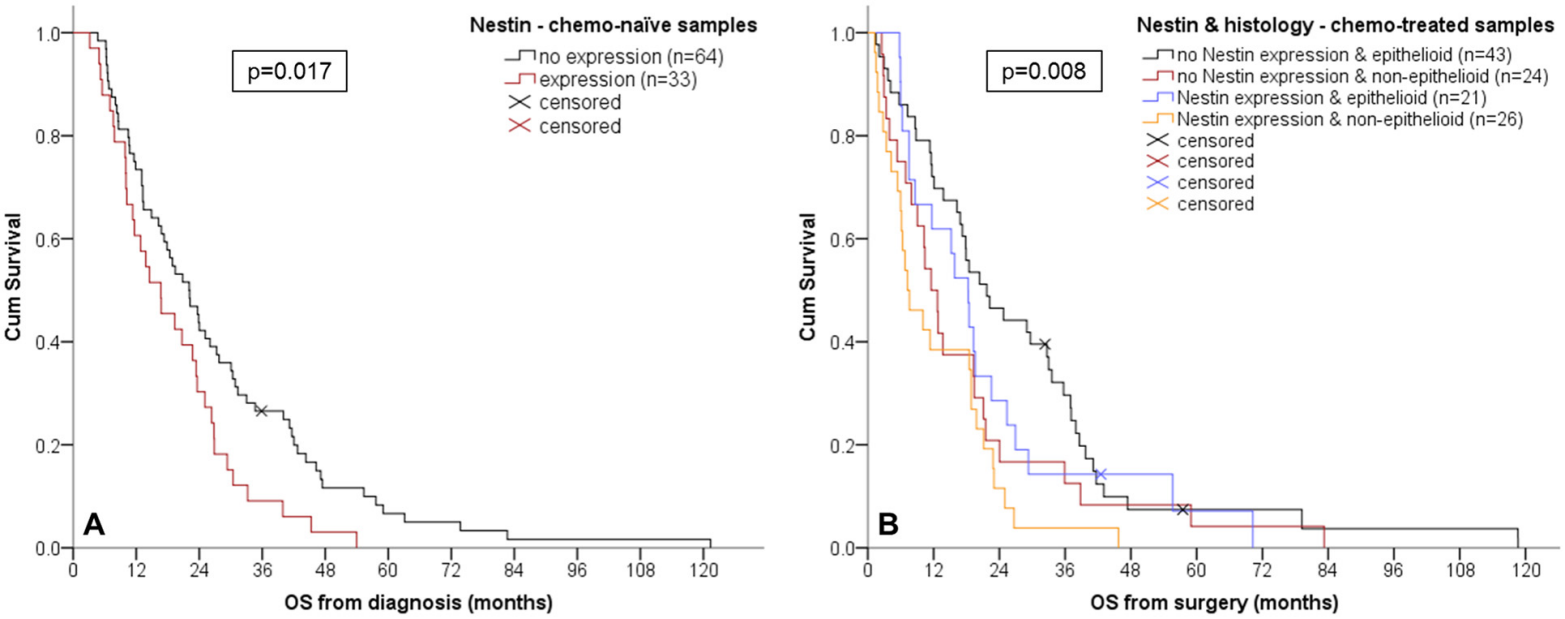
Kaplan-Meier curves. Survival curves presented for nestin expression in chemo-naïve biopsies (A) and the combination of nestin and histology in chemo-treated surgical specimens (B).

The Kaplan-Meier curves for nestin expression in chemo-naïve biopsies and the combination of nestin and histology in chemo-treated surgical specimens are presented in Fig 4.

Finally, the multivariate analysis of categorized histology together with marker expression (Table 4) revealed that histology and nestin expression are independent prognosticators of survival in chemo-naïve biopsies whereas only histology remains an independent prognosticator in chemo-treated surgical specimens

**Table 4:** Multivariate analysis of overall survival.

	HR (95% CI)	p
**Chemo-naïve biopsies (n = 97)**		
Histotype		*<0*.*001*
Biphasic vs. epithelioid	1.8 (1.1–2.8)	*0*.*023*
Sarcomatoid vs. epithelioid	13.9 (4.5–43.1)	*<0*.*001*
Nestin (expression vs. no expression)	1.6 (1.0–2.5)	*0*.*035*
Periostin (high (>20) vs. low (≤20) H-score)	1.2 (0.8–1.9)	n.s.
**Chemo-treated surgical specimens (n = 114)**		
Histotype		*0*.*012*
Biphasic vs. epithelioid	1.7 (1.1–2.5)	*0*.*014*
Sarcomatoid vs. epithelioid	2.3 (1.1–5.1)	*0*.*036*
Nestin (expression vs. no expression)	1.5 (1.0–2.2)	n.s.
Periostin (high (>75) vs. low (≤75) H-score)	1.4 (0.9–2.0)	n.s.

Categorized histology and dichotomized H-scores of nestin and periostin were included in the multivariate Cox regression analysis for both chemo-naïve biopsies and chemo-treated surgical specimens. HR: hazard ratio; CI: confidence interval.

## Discussion

In this study, we show that any expression of the neural crest stem cell factor nestin as well as high expression of the EMT protein periostin are associated with decreased overall survival of MPM patients when assessed by immunohistochemistry in chemo-naïve biopsies. In this setting, nestin proves to be an independent prognosticator against categorized histology. Further, any nestin expression in chemo-treated surgical specimens is also associated with decreased survival, a combination with non-epithelioid histology being particularly dismal. Neo-adjuvant chemotherapy using Pla/Pem increases the expression level of nestin in comparison to Pla/Gem, whereas the opposite is found for periostin. Nestin expression is significantly higher in biphasic and sarcomatoid MPM of the historic cohort 1 and in Pla/Pem treated biphasic MPM of the ITT cohort 2 in comparison to epithelioid tumors.

### Expression of the neural crest stem cell marker nestin in MPM

Herein, we found that most mesotheliomas (59–65%) were negative for nestin. In up to 5% we observed a strong staining (H-score of >201). According to the literature, a high nestin expression was observed in 25% (14/56) of primary melanomas, 50% (84/165) of melanoma metastases and 40% (21/53) of melanoma cell lines [19]. In serous ovarian carcinoma, nestin was detected in 33% of the tissues and its overexpression correlated with cisplatin-based CT resistance and shorter OS [20]. In MPM, any nestin expression was observed in 13% of the tumor cells of 6 epithelioid MPM [16].

Nestin is a class IV intermediate filament (IF) protein that copolymerizes into heteromeric filaments with class III IF proteins, mostly vimentin [11], thereby connecting the components of the cytoskeleton, coordinating changes in cell dynamics and contributing to vimentin disassembly during mitosis. Anchoring of the glucocorticoid receptor (GR) to colocalized cytosolic nestin-vimentin heterodimers was found to be related to high proliferation rate and poor prognosis [11, 21]. It may be interesting to study this mechanism in deciduoid mesothelioma which is particularly rich in vimentin filaments, as we have described previously [22]. Conceivably, the relative protein expression of both vimentin and nestin intermediate filaments may be related to biphasic/sarcomatoid differentiation and aggressiveness, respectively.

### Expression of stem cell and EMT markers in the histologic variants of MPM

We have previously investigated the EMT marker protein periostin on the MPM cohort 1 and have found it to be associated with the sarcomatoid variant [5]. This prompted us to test if a similar increase of the stem cell factor nestin exists along the EMT axis. In the historic cohort 1, an increase of nestin towards biphasic and sarcomatoid differentiation was found. In the ITT cohort 2, such an increase was significant only for Pla/Pem treated biphasic MPM compared to epithelioid. Statistics of the second cohort was restricted due to the low number of sarcomatoid MPM (n = 4 each for Pla/Gem and Pla/Pem neo-adjuvant CT, respectively). In conclusion, a transition of epithelioid to biphasic MPM may be associated with an increase of stem cell traits when applying certain types of CT. In a previous study we identified side population (SP) drug effluxing cells with self-renewal properties and increased chemoresistance among MPM cell lines and tumor-derived primary cell cultures [23]. Compared to the non-SP (NSP) fraction, the SP fraction led to development of mesotheliomas characterized by mesenchymal morphology and increased tumorigenicity. Other authors reported that non-epithelioid MPMs had lower E-cadherin but higher Snail, Twist and Zeb1 expression as well as a higher proliferation rate [24–25]. Transcriptome analysis identified two MPM subgroups C1/C2, whereby C2 included all sarcomatoid and desmoplastic cases with mesenchymal phenotype [26].

### EMT/SC mechanism in malignant pleural mesothelioma

EMT was proposed to be a mechanism for generation of cancer stem cells (CSCs) endowed with a more invasive and metastatic phenotype [2]. E.g. EMT of immortalized human mammary epithelia resulted in acquisition of mesenchymal and stem cell properties [27]. Such EMT-derived cells exhibited a multi-lineage differentiation potential similar to mesenchymal stem cells (MSC). Therefore, it was hypothesized that EMT programs emerge as important regulators of phenotypic plasticity in cancer cells, including their entrance into stem-cell states [28]. Yet, the frequency of CSCs in a given tumor and the definition of their stem-cell state, respectively, are highly debated. It is unclear if SC markers are restricted to *bona fide* CSCs that may be defined in a strict manner as being able to create an exact tumor phenocopy when transplanted from one mouse to the next, or if SC marker expression as assessed by IHC or RT-PCR extends into the cancer progenitor cell pool or even into the pool of differentiated cancer cells, reflecting rather histology plasticity with elaboration of stem cell traits.

The EMT concept has been investigated so far mostly for malignant epithelial tumors, thus by definition carcinomas and corresponding cell lines derived from them. In some organs like e.g. lung or bladder, sarcomatoid carcinomas may develop and are well recognized by their spindle cell phenotype. These tumors co-express cytokeratin and vimentin but generally they are rare and display a heterogeneous histology, mixed with adeno- or squamous cell carcinoma parts, making the analysis of the EMT impact on e.g. overall survival difficult. Clinically more frequent are biphasic tumors such as MPM, which are characterized by a constitutive intrinsic switch between an epithelial, called epithelioid, and a mesenchymal, called sarcomatoid, differentiation. Mesothelial cells are successors of the coelomic epithelium which originates from mesodermal mesenchyme via MET. The EMT transition from epithelioid to sarcomatoid MPM histology in turn reflects a reversion of embryological development. Thus, one may postulate that sarcomatoid MPM is more undifferentiated, similar to the embryonic mesodermal original tissue. In general, we believe that MPM is one of the best human in-vivo models to study the EMT/MET pathways.

### Effect of neo-adjuvant chemotherapy on marker expression

MPM displays a high intrinsic chemotherapy resistance with poor response rates <20%. Pla/Gem containing neo-adjuvant CT was administered in 59 patients, Pla/Pem in 85 patients, allowing for further statistical analysis. As platinum was included in both regimens, it is conceivable to attribute differences in immunoreactivity to Gem vs. Pem. The stem cell marker nestin and the EMT marker periostin showed a different alteration of immunoreactivity in response to these 2 regimens: Nestin expression increased, but periostin decreased in Pla/Pem compared to Pla/Gem chemotherapy.

The antimetabolite gemcitabine (Gemzar®) is a pyrimidine nucleoside analog which replaces cytidine during DNA replication and blocks the active site of ribonucleotide reductase (RNR). Gemcitabine-treated MPM tissues displayed higher periostin in comparison to the median of any chemotherapy (H-score 110 versus 75). This corroborates data on pancreatic carcinomas: Gem-resistant tumor cells were reported to display EMT features including spindle-shaped morphology, increased migration and reduced adhesion. Increase of vimentin as well as the SC markers CD24 and 44 was associated with decreased E-cadherin [29].

Pemetrexed (Alimta®) is a folate antimetabolite inhibiting thymidylate synthase, dihydrofolate reductase and glycinamide ribonucleotide formyltransferase, 3 enzymes of the purine and pyrimidine synthesis pathway. This drug is especially used for MPM CT. We have previously shown that neo-adjuvant CT, both Pla/Gem and Pla/Pem, induce senescence markers like p21 or plasminogen activator inhibitor-1 (PAI-1) in MPM [30]. A similar study reported the induction of SASP (senescence-associated secretory phenotype) by pemetrexed [31]. Thereby, conditioned media from senescent MPM triggered the emergence of EMT as well as clonogenic and chemoresistant cell subpopulations with high levels of ALDH (aldehyde dehydrogenase) activity. In here, we observed a higher expression of nestin after neo-adjuvant pemetrexed in comparison to gemcitabine, indicating that the former drug may be more effective in activating its expression.

This opens up the most intriguing question to what degree such induction chemotherapy creates a subsequent intrinsic chemotherapy resistance itself via induction of EMT/SC traits. At least for gemcitabine, one may hypothesize that this drug favours a fibrotic remodelling of the peritumoral stroma containing increased amounts of the non-structural matricellular N-glycoprotein periostin.

There was no significant association with response to induction chemotherapy using the computer tomography based RECIST criteria. The major RECIST parameter is the total tumor thickness measured perpendicular to the chest wall and the mediastinum, respectively. When evaluating surgical EPP specimens on whole sections from several tumor blocks, chemoresistance is often observed in MPM. Typically, vital areas of 50 to >90% of total tumor cells are intermingled with minor areas of focal response presenting as necrosis or foamy cell degeneration. As MPM is a multifocal disease, correlation of marker expression with response would most likely need precise topographic mapping between hot/cold spots on positron-emission tomography/computer tomography (PET/CT) and histologic sectioning at the very same position. Non-tumoral inflammatory fibrosis and giant-cell reaction to talc pleurodesis will although also contribute to glucose uptake.

In summary, our study shows that the stem cell factor nestin is a prognosticator for overall survival of MPM patients and may allow for further stratification together with the histologic variant.
